# Antioxidants and Oxidants in Boar Spermatozoa and Their Surrounding Environment Are Associated with AMPK Activation during Liquid Storage

**DOI:** 10.3390/vetsci10030214

**Published:** 2023-03-10

**Authors:** Junwei Li, Wenming Zhao, Jiaqiao Zhu, Huiming Ju, Ming Liang, Shuaibiao Wang, Shufang Chen, Graça Ferreira-Dias, Zongping Liu

**Affiliations:** 1 College of Veterinary Medicine, Yangzhou University, Yangzhou 225009, China; lijunwei@yzu.edu.cn (J.L.); zhaowenming2018@126.com (W.Z.); jqzhu1998@163.com (J.Z.); hmju@yzu.edu.cn (H.J.); 2 Jiangsu Co-Innovation Center for Prevention and Control of Important Animal Infectious Diseases and Zoonoses, Yangzhou University, Yangzhou 225009, China; 3 Department of Feeding Microecology, Shandong Baolaililai Bioengineering Co., Ltd., Tai’an 271001, China; liangming@blll1004.onexmail.com; 4 DanAg Agritech Consulting (Zhengzhou) Co., Ltd., Zhengzhou 450000, China; billwang@danagintl.com; 5 Royal Veterinary College, London NW1 0TU, UK; 6 Ningbo Academy of Agricultural Science, Ningbo 315040, China; jhynku@163.com; 7 CIISA-Centre for Interdisciplinary Research in Animal Health, Faculty of Veterinary Medicine, University of Lisbon, 1300-477 Lisbon, Portugal; gmlfdias@fmv.ulisboa.pt; 8 Associate Laboratory for Animal and Veterinary Sciences (AL4AnimalS), 1300-477 Lisbon, Portugal

**Keywords:** boar semen, liquid storage, AMPK activation, antioxidants

## Abstract

**Simple Summary:**

Liquid storage of boar semen at 17 °C is a conventional method used for artificial insemination in pig reproduction. During storage, boar sperm quality continuously decreases with time, which compromises the field results after artificial insemination. Oxidative stress induced by storage is considered as the main cause of the quality loss. It is crucial to have a better understanding of the mechanism of sperm resistance to oxidative stress. The AMP-activated protein kinase was found in boar spermatozoa and plays a positive role in maintaining sperm quality and functionality when activated. This study aimed to explore if AMP-activated protein kinase could be activated when spermatozoa suffer oxidative stress. Our results show that sperm quality decreased with storage time, which correlated to antioxidant and oxidant levels in spermatozoa and their surrounding environment that was correlated to the activation of the AMP-activated protein kinase. Treatment of boar semen with H_2_O_2_ (one of the reactive oxygen species) confirmed the positive role of oxidative stress in activating the AMP-activated protein kinase. Taken together, oxidative stress, reflected by the predominance of oxidants over antioxidants in boar semen, promotes activation of the AMP-activated protein kinase. The findings shed light on the mechanism of sperm resistance to OS and provide information for selecting antioxidants added into semen extender for better reproductive results.

**Abstract:**

Activation of the AMP-activated protein kinase (AMPK) has been demonstrated to be beneficial for boar sperm quality and functionality, while the underlying mechanism of AMPK activation of boar spermatozoa remains obscure. This study aimed to explore the effect of antioxidants and oxidants in boar spermatozoa and their surrounding fluid (SF) on the activation of AMPK during the liquid storage. Ejaculates from Duroc boars, routinely used for semen production, were collected and diluted to a final concentration of 25 × 10^6^/mL. In experiment 1, twenty-five semen samples from eighteen boars were stored at 17 °C for 7 days. In experiment 2, three pooled semen samples created from nine ejaculates of nine boars were used, and each sample was treated with 0, 0.1, 0.2, and 0.4 μM/L H_2_O_2_ and stored at 17 °C for 3 h. Sperm quality and functionality, antioxidants and oxidants in boar spermatozoa and SF, the intracellular AMP/ATP ratio, and the expression levels of the phosphorylated AMPK (Thr172) were determined. Sperm quality significantly decreased with storage time in terms of viability (*p* < 0.05). Antioxidant and oxidant levels were markedly affected with storage time, with a decline in the SF total antioxidant capacity (TAC) (*p* < 0.05), SF malondialdehyde (MDA) (*p* < 0.05), and the sperm’s total oxidant status (TOS), as well as a fluctuation in sperm superoxidase dismutase-like (SOD-like) activity (*p* < 0.05). The intracellular AMP/ATP ratio increased (*p* < 0.05) on day 4 and subsequently decreased to its lowest value on days 6 and 7 (*p* < 0.05). The phosphorylated AMPK levels increased from day 2 to day 7 (*p* < 0.05). Correlation analyses indicate that sperm quality during liquid storage was correlated to antioxidants and oxidants in spermatozoa and SF (*p* < 0.05), which were correlated to the phosphorylation of sperm AMPK (*p* < 0.05). Treatment with H_2_O_2_ induced damages in sperm quality (*p* < 0.05), a decline in antioxidant levels (SF TAC, *p* < 0.05; sperm SOD-like activity, *p* < 0.01), an increase in oxidant levels (SF MDA, *p* < 0.05; intracellular ROS production, *p* < 0.05), a higher AMP/ATP ratio (*p* < 0.05), and phosphorylated AMPK levels (*p* < 0.05) in comparison with the control. The results suggest that antioxidants and oxidants in boar spermatozoa and SF are involved in AMPK activation during liquid storage.

## 1. Introduction

Artificial insemination (AI) has been a conventional tool for reproduction in the porcine industry. Extended semen is widely utilized for AI [1], which offers the best use of boar ejaculates and tremendously reduces the cost. However, a continuous decline in sperm quality and functionality is observed during the storage of AI doses [2], which compromises the fertility results in the field. The excessive production of reactive oxygen species (ROS) induces oxidative stress (OS), which has been considered as the main factor that deteriorates sperm quality and functionality [3] during the freezing–thawing process and liquid storage [4,5]. Our previous study indicated that certain enzymatic and non-enzymatic antioxidants in seminal plasma are involved in sperm resistance to cryopreservation by playing against OS, including glutathione peroxidase 5 (GPX5), superoxidase dismutase (SOD), and low-weight molecules with antioxidant properties in terms of the total antioxidant capacity (TAC) and ferric-reducing ability of plasma [4]. Relations between those antioxidants in seminal plasma and sperm parameters were reported in buffalo bull [6], bull [7], avian [8], canine [9], and equine [10]. Favorable results with certain antioxidants or their combinations supplemented into the semen extender confirm the beneficial impact of antioxidants on semen storage [11]. In fact, a redox balance is crucial for sperm quality and functionality. As is well known, the excessive generation of ROS causes OS, which exerts harmful effect on spermatozoa via the peroxidation of lipids, the induction of oxidative DNA damage, and the formation of protein adducts. Meanwhile, the physiological levels of ROS participate in the process of sperm capacitation [12]. Thus, the type and quantity of antioxidants should be considered when they are applied to protect spermatozoa from OS. Therefore, it is important to consider how spermatozoa regulate the redox balance.

To answer the above-mentioned question, we may have to explain how spermatozoa resist OS. During the process of sperm biotechnology, once OS occurs, the stress stimulator forms and subsequently spermatozoa initiate self-adjustments, one of which is the activation of the AMP-activated protein kinase (AMPK) [13]. The AMPK plays a vital role in balancing cell metabolism and was first found in mammalian spermatozoa in 2012 [14,15]. The activation of the AMPK exerts a positive effect on sperm quality and functionality, while the inhibition of the AMPK imposes negative impacts [15,16,17,18]. This raises the question based on how the AMPK is activated in spermatozoa. Evidence in somatic cells shows that the AMPK can be activated by its upstream kinases, i.e., liver kinase B1 (LKB1) and Ca^2+^ calmodulin-dependent kinase 2 (CaMKK2), by an increase in the AMP/ATP ratio [19], and indirectly by mitochondria-derived ROS by influencing ATP production [20]. In mammalian spermatozoa, so far, there is no direct evidence of the activation of the AMPK by LKB-1 or CaMKK2. It has been reported that the soluble adenylate cyclase (sAC), cAMP, and the cAMP-dependent protein kinase (PKA)-mediated pathway lie upstream of AMPK activation in boar spermatozoa [14,16,21]. The cell stimulus that increases cAMP levels could promote sperm AMPK activation through cAMP degradation to AMP by phosphodiesterases, as occurs in somatic cells [22]. Intracellular Ca^2+^ has been demonstrated as an upstream regulator of AMPK activation by activating the specific sperm sAC and its downstream signaling through the PKA in boar spermatozoa [16,21], or via the activation of CaMKKs that is proven to exist in chicken spermatozoa [23]. Further studies are needed to explore how the AMPK is activated during boar semen storage, which in turn helps to elucidate the mechanism of sperm resistance to OS.

Based on the current evidence, we hypothesized that antioxidants and oxidants in boar spermatozoa and their surrounding environment contribute to sperm survival; an imbalance of antioxidants and oxidants is associated with the activation of the sperm AMPK. To confirm this hypothesis, we investigated the influence of antioxidants and oxidants on boar sperm quality and functionality, as well as AMPK activation during the long-term storage of boar semen at 17 °C. Sperm samples were further treated with H_2_O_2_ to confirm the links between antioxidants and oxidants, as well as the activation of the sperm AMPK. The findings were expected to shed light on the mechanism of sperm resistance to OS and to provide information for selecting antioxidants added into semen extender for better reproductive results.

## 2. Materials and Methods

### 2.1. Semen Handling and Experiment Design

Ejaculates used in this study were purchased from two commercial AI stations (Henan Swinegenes Co., Ltd., Hebi, China; Shanghai Sunsing Co., Ltd., Shanghai, China). The boars used for semen collection were healthy, mature, and fertile; they were also housed in AI stations under environmentally controlled conditions and were given commercial feed, according to semen donor requirements. Ejaculates were collected using the gloved-hand method and diluted to 25 × 10^6^ sperm/mL with the ACROMAX PLUS extender (ZoitechLab S.L., Madrid, Spain). Semen samples were packaged in a well sealed foam box together with ice bags and were delivered to Yangzhou University in approximately 30 h. A temperature monitor previously deposited in the foam box showed a temperature change within 17–22 °C during delivery. Once arrived, the semen quality was evaluated, and only samples with sperm motility > 70%, sperm viability > 70%, and abnormality < 15% were selected for the experiments. In experiment 1, twenty-five ejaculates from eighteen Duroc boars were used. The semen samples were stored at 17 °C for seven days, and sperm quality and functionality were measured daily. One part of semen samples from each day was taken out and centrifuged twice (2400× *g*, 3 min, 17 °C) to separate spermatozoa and their surrounding fluid (SF, a mixture of extender and seminal plasma). The obtained SF was further centrifuged three times (2400× *g*, 3 min, 17 °C) and was microscopically examined to confirm that it was sperm-free and then stored at −80 °C until oxidant and antioxidant analyses were performed. The harvested sperm samples were washed three times with PBS by centrifugation (2400× *g*, 3 min, 17 °C) and stored at −80 °C for the determination of AMPK phosphorylation, the ATP assay, and antioxidant and oxidant measurements. In experiment 2, nine ejaculates from nine Duroc boars were pooled to create three semen samples (one semen sample was made of three ejaculates to diminish variations in ejaculates). Thereafter, the semen samples were divided into four groups, and then they were treated with 0, 0.1, 0.2, and 0.4 μM/L H_2_O_2_. After incubation at 17 °C for 3 h, the sperm quality, intracellular ROS level, antioxidant and oxidant levels in boar spermatozoa and SF, intracellular ATP assay, and sperm AMPK phosphorylation were determined.

### 2.2. Assessment of Sperm Quality and Functionality

Sperm motility was objectively evaluated using an integrated sperm analysis system (ISASV1^®^; Proiser R + D, Paterna, Spain). Briefly, a 5 µL semen sample (25 × 10^6^ sperm/mL) was placed in a Makler counting chamber (Sefi Medical Instruments, Haifa, Israel), and it was pre-warmed to 38 °C. Then, four to five fields were captured to analyze a minimum of 400 spermatozoa per sample. Sperm total motility was recorded as the percentage of total motile spermatozoa (with an average path velocity ≥20 µm/s) and progressive motility as the percentage of sperm exhibiting rapid and progressive movement (straight line velocity ≥ 40 µm/s).

A flow cytometer (CytoFLEX S, Beckman Coulter Inc., Brea, CA, USA) was used to evaluate the sperm viability, mitochondrial membrane potential, and intracellular ROS production. 

The sperm viability was evaluated in terms of plasma and acrosomal membrane integrity using a triple-fluorescence procedure [24]. Briefly, a 100 μL sperm sample (25 × 10^6^ sperm/mL) was incubated with 3 μL H-42 (Hoechst 33342, B2261, Sigma, 0.05 mg/mL in PBS), 2 μL PI (propidium iodide, P3566, Thermofisher, 0.5 mg/mL in PBS), and 2 μL PNA-FITC (L7381, Sigma, 200 μg/mL in PBS) at 37 °C in the dark for 10 min. Before analysis via flow cytometry, 400 μL PBS was added to each sample. The sperm viability was expressed as a percentage of live spermatozoa with a negative PI and a negative PNA-FITC. The sperm population with a negative PI and a positive PNA-FITC was regarded as viable spermatozoa with a damaged acrosome membrane. 

The mitochondrial membrane potential was evaluated with Mitotracker Deep Red 633 (M22426, Thermofisher, Waltham, MA, USA), using a protocol described by Alkmin et al. (2014) [25], with slight modification. Briefly, a 100 μL sperm sample (25 × 10^6^ sperm/mL) was transferred to culture tubes containing 3 μL of H-42 (0.05 mg/mL in PBS), 2 μL of PI (0.5 mg/mL in PBS), and 5 μL of Mitotracker (0.2 μM in PBS of a stock solution of 1 mM in DMSO). The samples were incubated at 37 °C in the dark for 15 min. Before flow cytometry analysis, 400 μL of PBS was added to each sample. The data were recorded as the percentage of viable spermatozoa with a high mitochondria membrane potential (negative PI and positive Mitotracker).

The intracellular generation of ROS in the viable spermatozoa was measured using CM-H2DCFDA (C6827, Thermofisher), following the procedure described by Guthrie and Welch [26]. Briefly, a 50 μL semen sample (25 × 10^6^ sperm/mL) was re-diluted in 950 μL of PBS containing 1.5 μL of H-42 (0.05 mg/mL in PBS), 1 μL of PI (0.5 mg/mL in PBS), and 1 μL of CM-H2DCFDA (1 mM in DMSO), and 1 μL of TBH (458139, Sigma, St. Louis, MO, USA, 70% in distilled water) was added to the positive control group. Then, the samples were incubated at 37 °C in the dark for 30 min prior to flow cytometric analysis. The data were recorded as fluorescence units per million viable spermatozoa with a high intracellular ROS generation (PI-negative and DCF-positive).

### 2.3. Measurement of Antioxidants and Oxidants in Boar Spermatozoa and SF

Colorimetric methods were carried out to measure antioxidants and oxidants in boar spermatozoa and SF using a micro-plate reader (PowerWave XS; Bio-Tek Instruments, Winooski, VT, USA). SF samples were directly used for assays. Sperm samples containing 30 × 10^6^ cells were centrifuged (1600× *g*, 5 min) and resuspended with 360 μL 1% Triton X-100 to lyse cells at 4 °C for 20 min. Thereafter, the supernatant was collected by centrifugation (4000× *g*, 30 min, 4 °C) and kept on ice for assays.

The total oxidant status (TOS) was determined, as described by Erel [27]. Oxidants present in the sample oxidize the ferrous ion–chelator complex to ferric ion. The oxidation reaction is prolonged by glycerol, which is abundantly present in the reaction medium. The ferric ion makes a colored complex with chromogen in an acidic medium. The color intensity, which can be measured spectrophotometrically, is related to the total amount of oxidant molecules present in the sample. The results were expressed in terms of micromolar hydrogen peroxide equivalent per liter (μM H_2_O_2_ Equiv./L). The malondialdehyde (MDA) contents were evaluated using a commercial kit (A003-1-2, Nanjing Jiancheng, Nanjing, China), following the manufacturer’s instructions. The MDA in the sample reacts with thiobarbituric acid (TBA) to generate a MDA-TBA adduct, which can be quantified colorimetrically (OD = 532 nm). The MDA content results were expressed in nM/mL. The total antioxidant capacity (TAC) was measured in terms of the Trolox-equivalent antioxidant capacity using a commercial kit (S0121, Beyotime, Shanghai, China). The method used is based on 2, 2′-azinobis-(3-ethylbenzothiazoline-6-sulfonate) decolorization by antioxidants, according to their concentration and antioxidant capacity. The change is detected at 414 nm using a microplate reader. The results of the TAC assay were expressed in mM/L. 

SOD-like activity determination was performed using a commercial kit (A001-3-2, Nanjing Jiancheng, Nanjing, China). The assay is based on the capacity of SOD-like antioxidants to catalyze the dismutation of the superoxide anion, produced by the action of xanthine oxidase, into hydrogen peroxide and oxygen. As superoxide anions act on WST-1 to produce a water-soluble formazan dye that can be detected by an increase in absorbance at 450 nm, the greater the SOD-like activity in the sample, the less formazan is produced. A 50% SOD-like antioxidant inhibition corresponds to the SOD-like activity of 1 U. The SOD-like activity was calculated from the Equation:

$$SOD–like\;antioxidants\;inhibition\left(\%\right)=\frac{\left(A\;blank1-A\;blank3)-(A\;sample-A\;blank2\right)}{\left(A\;blank1-A\;blank3\right)}$$

$$SOD–like\;activity\left(U/mL\right)=\frac{SOD–like\;antioxidants\;inhibition}{50\%}\times Dilution\;ratio$$

The intracellular GPX5-like activity was determined using an enzyme-linked immune bi-antibody sandwich two-step method with a commercial kit (ml622032, Shanghai Enzyme Link, Shanghai, China). After the incubation of samples in wells pre-coated with GPX5 capture antibody, HRP-labeled detection antibody was added and thereafter washed. TMB was used as a substrate for color development. GPX5-like activity in samples was reflected by the color change in the TMB catalyzed by GPX5, which was detected at 450 nm using a microplate reader. The results were expressed as U/mL.

### 2.4. Determination of Intracellular ATP, ADP, and AMP Content

The assays for intracellular ATP, ADP, and AMP were performed, as described by Nguyen et al. [28]. Briefly, the sperm samples of 100 µL (20 × 10^6^ cells/mL) were mixed with 1 µL of phosphatase inhibitor cocktail (P002, NCM Biotech, Suzhou, China) and kept for 30 min on ice. Thereafter, tubes containing 900 µL of boiling buffer (50 mM Tricine, 10 mM MgSO4, 2 mM EDTA, pH 7.8) were heated for 5 min at 95 °C. After the addition of samples, the mixture was heated for 10 min at 95 °C. The tubes were chilled on ice for 10 min and then centrifuged at 5000× *g* for 30 min at 4 °C (5810 R, Eppendorf, Hamburg, Germany). The supernatant was used for assays. Three aliquots (100 µL for each) of target sperm samples were incubated with 25 µL of Buffer A (75 mM Tricine, 5 mM MgCl_2_, and 0.0125 mM KCl, pH 7.5), Buffer B (Buffer A + 0.1 mM phosphoenolpyruvate (P7002, Sigma-Aldrich, St. Louis, MO, USA) + 0.08 µg/µL of the pyruvate kinase (P1506, Sigma-Aldrich)), and Buffer C (Buffer B + 0.1 µg/µL of the adenylate (myo) kinase (M3003, Sigma-Aldrich)). Tubes containing Buffers A, B, and C were incubated at 30 °C for 30 min, 30 min, and 90 min, respectively. All three tubes were boiled at 95 °C for 3 min to stop reactions and then chilled on ice until they were assayed for ATP content. Buffer A was used to measure ATP content in the sperm samples. Buffer B was used to determine the combined amount of ATP and ADP according to reaction 1, in which ADP is transformed to ATP with the pyruvate kinase as a catalytic agent. Buffer C was used to measure the combined amount of ATP, ADP, and AMP according to reaction 2 (in which AMP was converted into ADP with the adenylate kinase) and reaction 1 (in which ADP was converted into ATP). The ATP content in each sample aliquot was measured using a commercial kit (FLAA, Sigma), following the manufacturer’s instructions. The ADP and AMP content was calculated based on the reaction formulas. The results are expressed as pM/L.

(1) $$ADP+Phosphoenolpyruvat\overset{pyruvate\;kinase}{\to }ATP+Pyruvate$$

(2) $$AMP+ATP\overset{adenylate\;kinase}{\to }2ADP$$

### 2.5. Western Blotting

Samples containing 1.2 × 10^8^ sperm cells were centrifuged at 2400× *g* for 3 min at 4 °C. Thereafter, they were washed with PBS and re-suspended with RIPA buffer containing 1% protease and phosphatase inhibitor cocktail (EDTA-Free, 100X in DMSO) for 10 min at 4 °C. The samples were lysed by ultrasonication (20 KHz, 750 W, operating at 30% power, six cycles for 5 s on and 5 s off). After 30 min of lysis at 4 °C, the samples were centrifuged at 12,000× *g* for 10 min at 4 °C. A portion of the supernatant was used to analyze the concentration of total protein, while the rest was mixed with 5 × SDS loading buffer and boiled at 95 °C for 10 min. The proteins were separated by 10% SDS-PAGE at 90 V for 120 min, and they were subsequently transferred onto PVDF membranes at 220 mA for 90 min. Western blotting analyses were performed using anti-phospho-Thr172 AMPKα (2535S, Cell Signaling Technology, Danvers, MA, USA, 1:1000), anti-total AMPKα2 (ab231807, Abcam, Cambridge, UK, 1:1000), and anti α-tubulin (AF0001, Beyotime, Shanghai, China, 1:1000) as primary antibodies. The membrane signal was acquired with an automatic chemiluminescence image analysis system (Tannon 5200). Image analysis was performed using ImageJ software (Image, Inc., Beverly Hills, CA, USA).

### 2.6. Statistical Analysis

The IBM SPSS software (version 19.0) was used for data analyses. The normality of the data set was tested based on the residuals using the Kolmogorov–Smirnov test. The statistical significance of differences in mean values between two groups was analyzed using unpaired *t* test. The one-way ANOVA or the Kruskal–Wallis one-way ANOVA were used where appropriate to perform a comparison among three or more groups, followed by LSD multiple-comparison tests. Pearson correlation analysis was utilized to evaluate the links between the phospho-AMPK level, the sperm quality and functionality, the antioxidant and oxidant levels in boar spermatozoa and their surrounding environment, and the intracellular AMP/ATP ratio. The values are expressed as the means ± SEMs. A *p* value < 0.05 was considered as the minimal level of significance.

## 3. Results

### 3.1. Boar Sperm Quality and Functionality Deteriorated with Liquid Storage Time at 17 °C

Changes in boar sperm quality and functionality are presented in Table 1. During liquid storage at 17 °C for seven days, no significant changes in boar sperm total and progressive motility were observed (*p* > 0.05). Sperm viability was significantly decreased (*p* < 0.05) with extended storage time, while no significant damage in acrosomal membrane of viable sperm was observed. The intracellular ROS level was found to be the highest (*p* < 0.05) on the first day of storage, was significantly reduced at day two (*p* < 0.05), and then was slightly increased until a significant decline (*p* < 0.05) was observed on days six and seven. The mitochondrial membrane potential was lower on day six than that on days two and five (*p* < 0.05).

### 3.2. Effects of Liquid Storage Time at 17 °C on Antioxidant and Oxidant Levels in Boar Spermatozoa and SF

**Figure 1:**
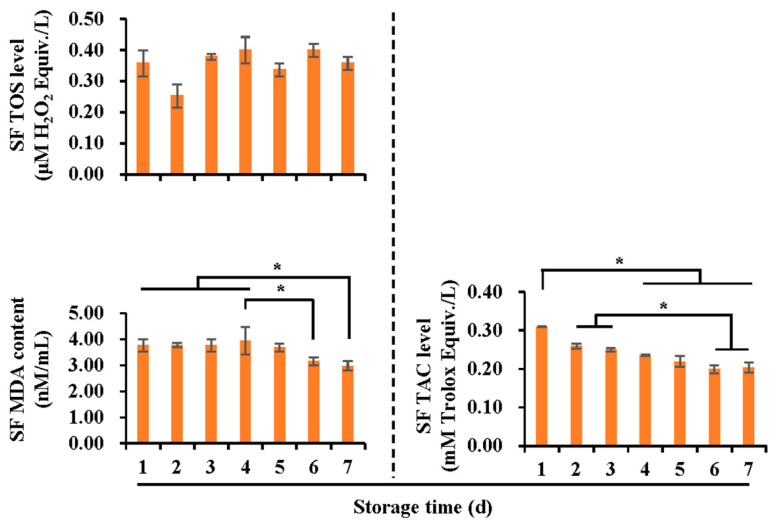
Histograms showing antioxidant and oxidant levels in SF (sperm surrounding fluid), according to storage time (days). TOS: total oxidant status, MDA: malondialchehyche, TAC: total antioxidant capacity. Data were pooled from 18 Duroc boars and are expressed as the mean ± SEM. * indicates differences among groups, *p* < 0.05.

**Figure 2:**
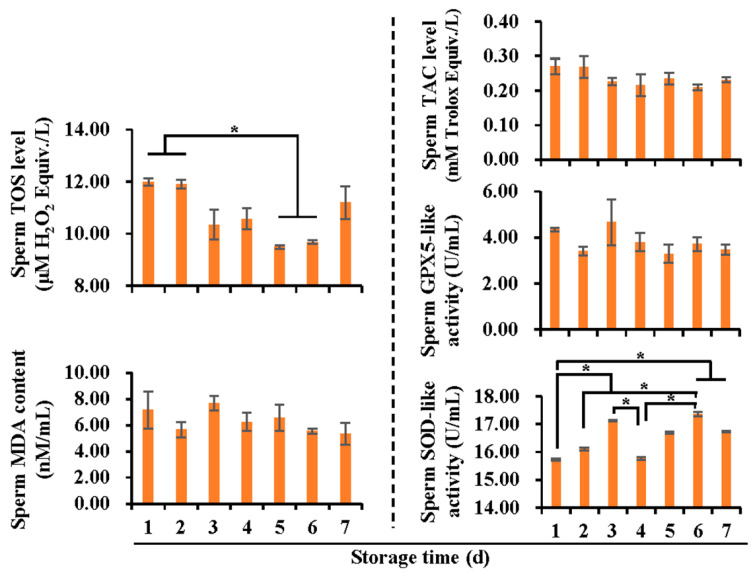
Histograms showing antioxidant and oxidant levels in spermatozoa, according to storage time (days). TOS: total oxidant status, MDA: malondialchehyche, TAC: total antioxidant capacity, GPX5: glutathione peroxidase 5, SOD: superoxide dismutase. Data were pooled from 18 Duroc boars and expressed as mean ± SEM. * indicates differences among groups, *p* < 0.05.

To determine the effect of oxidative stress on boar semen storage, the oxidants and antioxidants in both SF (Figure 1) and spermatozoa (Figure 2) were evaluated in terms of non-enzymatic and enzymatic substances. In SF, no significant changes in TOS levels were observed, while a significant decline (*p* < 0.05) in MDA was found after five days of storage. The TAC levels decreased (*p* < 0.05) after three days of storage, being the lowest (*p* < 0.05) on day six and seven. In spermatozoa, intracellular TOS level was lower (*p* < 0.05) on day five and six of storage, compared with that at days one and two. SOD activity increased (*p* < 0.05) from day one to day three, decreased on day four (*p* < 0.05), and increased subsequently to be the highest on day six (*p* < 0.05). No significant changes in intracellular TAC, GPX5, and MDA levels were found.

### 3.3. Effects of Liquid Storage Time at 17 °C on Expression of the Phosphorylated AMPK and the Intracellular AMP/ATP Ratio

**Figure 3:**
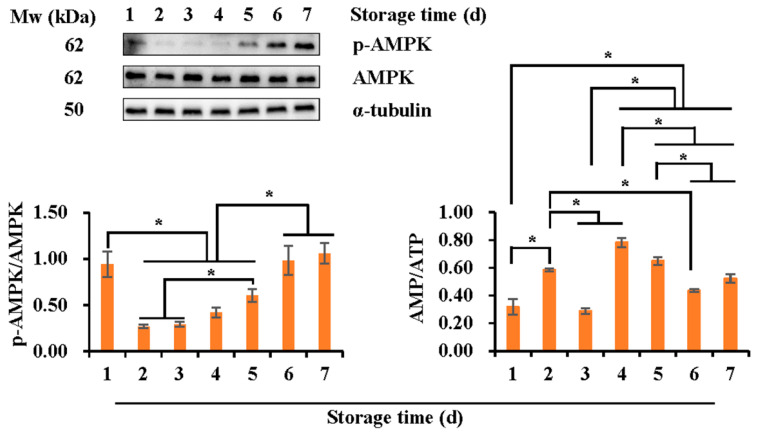
Expression of the phosphorylated AMPK and the intracellular AMP/ATP ratio during liquid storage at 17 °C for seven days. All Western blots were representative of three biological replicates. Bar graphs stand for data expressed as mean ± SEM, pooled from 18 Duroc boars. * indicates differences among groups, *p* < 0.05 (please find the WB full membrane in Figure S1).

As shown in Figure 3, the expression of the phosphorylated AMPK increased (*p* < 0.05) with storage time ranging from day two to seven, while it was higher on the first day of storage than that on days two and three (*p* < 0.05). During liquid storage, a great increase in the AMP/ATP ratio occurred on day four, being higher (*p* < 0.05) than that of the first three days and followed by a reduction to be lowest (*p* < 0.05) on days six and seven. Notably, an increase (*p* < 0.05) from day one to day two was observed. 

### 3.4. Correlation Analyses

Correlation analysis was performed to explore the effect of antioxidants and oxidants, as well as the AMP/ATP ratio, on boar sperm quality during liquid storage. Sperm viability was correlated, respectively, to SF TAC (R = 0.756, *p* < 0.05), SF MDA (R = 0.584, *p* = 0.168), sperm TOS (R = 0.419, *p* = 0.350), and the AMP/ATP ratio (R = −0.487, *p* = 0.268). 

To discover the links between AMPK phosphorylation and antioxidant and oxidant levels in boar spermatozoa and SF, correlation analysis was performed. The phosphorylated AMPK level was correlated to SF MDA (R = −0.453, *p* < 0.05). The AMP/ATP ratio was correlated to SF TAC (R = −0.339, *p* = 0.133).

### 3.5. Effect of H_2_O_2_ Treatment on Sperm Quality

**Figure 4:**
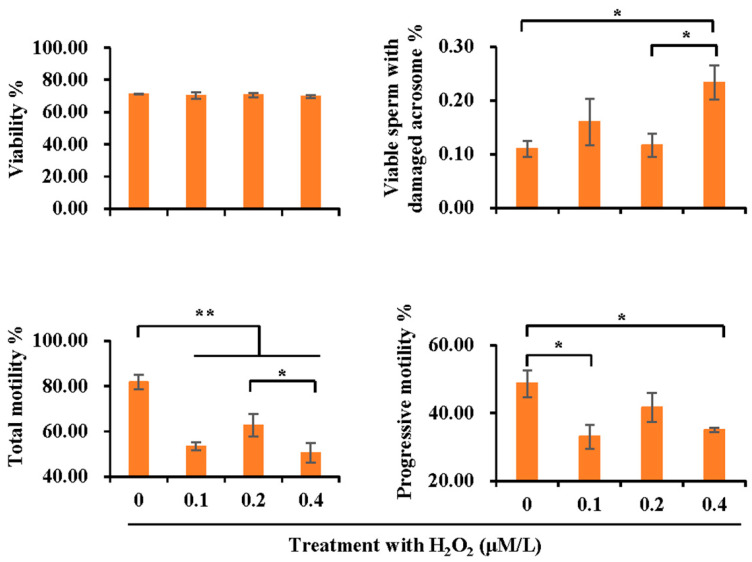
The effect of H_2_O_2_ treatment (0, 0.1, 0.2 and 0.4 μM/L) on sperm quality in terms of viability, acrosome integrity, total motility, and progressive motility. Bar graphs represent data expressed as mean ± SEM. * indicates differences among groups, *p* < 0.05; **, *p* < 0.01.

To confirm the effect of antioxidants and oxidants in boar spermatozoa and SF on sperm quality and AMPK activation, semen samples were treated with different concentrations of H_2_O_2_. Compared with the control, treatment with H_2_O_2_ greatly affected sperm quality (Figure 4), showing damages in sperm acrosome integrity (*p* < 0.05), total motility (*p* < 0.01), and progressive motility (*p* < 0.05). No alteration in sperm viability was observed.

### 3.6. Effect of H_2_O_2_ Treatment on Antioxidant and Oxidant Levels in Boar Spermatozoa and SF

**Figure 5:**
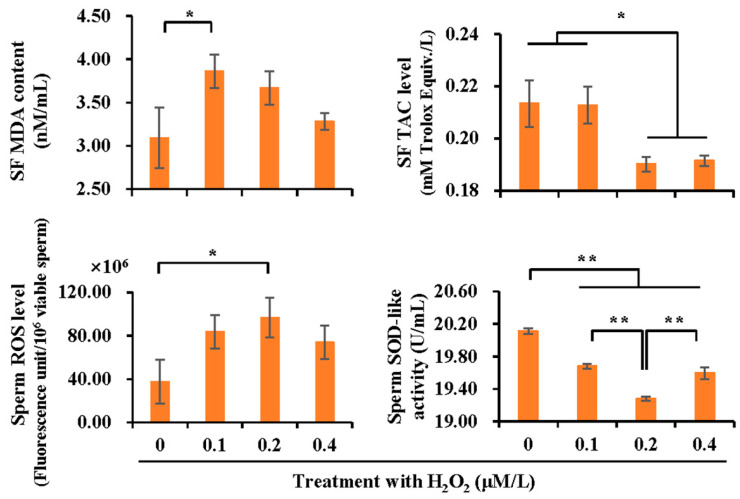
The effect of H_2_O_2_ treatment (0, 0.1, 0.2 and 0.4 μM/L) on antioxidant and oxidant levels in boar spermatozoa and SF. MDA: malondialchehyche, ROS: reactive oxygen species, TAC: total antioxidant capacity, SOD: superoxide dismutase. Bar graphs stand for data expressed as mean ± SEM. * indicates differences among groups, *p* < 0.05; **, *p* < 0.01.

Treatment with H_2_O_2_ induced higher levels of SF MDA (*p* < 0.05) and intracellular ROS production (*p* < 0.05), lower levels of SF TAC (*p* < 0.05), and lower sperm SOD-like activity (*p* < 0.01) than those of the control (Figure 5).

### 3.7. Effect of H_2_O_2_ Treatment on AMPK Phosphorylation and AMP/ATP Ratio of Boar Spermatozoa

**Figure 6:**
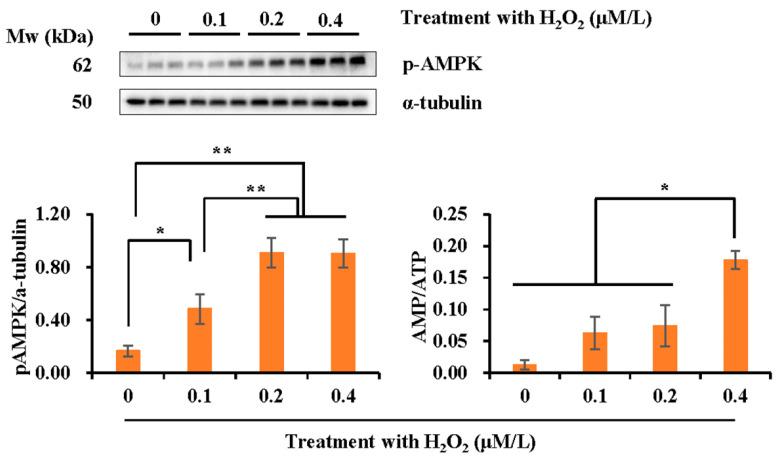
The effect of H_2_O_2_ treatment (0, 0.1, 0.2, and 0.4 μM/L) on the expression of the phosphorylated AMPK and the intracellular AMP/ATP ratio. All the Western blots are representative of three biological replicates. Bar graphs represent data expressed as mean ± SEM. * indicates differences among groups, *p* < 0.05; ** *p* < 0.01 (please find the WB full membrane in Figure S2).

As shown in Figure 6, higher AMPK phosphorylation levels were found in semen samples treated with 0.1 μM/L H_2_O_2_ (*p* < 0.05) and 0.2–0.4 μM/L H_2_O_2_ (*p* < 0.01) compared to that in the control group. The AMP/ATP ratio was higher in semen samples treated with 0.4 μM/L H_2_O_2_ than that in the rest groups (*p* < 0.05).

## 4. Discussion

The liquid storage of boar semen at 16–18 °C has been widely accepted as a conventional technique used for efficient porcine reproduction. However, a continuous decline in sperm quality and functionality has been observed during storage [29]. Factors, such as boar ejaculate fraction, extender type, and storage time, influence semen quality [30]. In this study, sperm viability was altered by liquid storage time, which is consistent with the results reported by Dziekonska and collaborators [31]. As boar spermatozoa are rich in polyunsaturated fatty acids, they are extremely susceptible to OS [32]. OS is the main cause of membrane peroxidation and consequently deteriorates viability. In the present study, the significantly high level of intracellular ROS at day 1 is probably due to a delivery of 30 h in a sealed foam box and temperature changes during delivery (17 °C at initial and about 22 °C at arrival). After being transferred to a refrigerator and stored at 17 °C, the metabolic activity of spermatozoa decreases, which reduces the production of ROS. In addition, substances with antioxidant properties in boar semen play against ROS, which further decreases the intracellular ROS. With increased storage time, the intracellular ROS level increased from day two to day five. The decline in the ROS level in spermatozoa on day six and day seven could be attributed to the compromised sperm quality in terms of decreased viability and mitochondrial membrane potential, as mitochondria are the main place for ROS production. A high level of intracellular ROS production at the beginning of storage could be responsible for the damage in viability with storage time, which stresses the importance of ROS monitoring during the liquid storage of boar semen, as suggested by Khoi et al. [5]. In addition, our results show that the mitochondrial membrane potential of spermatozoa decreased significantly on day six, when sperm viability and intracellular ROS production declined. Under physiological conditions, the byproduct of peroxidation caused by ROS enhances the production of ROS in mitochondria [3]. In the present study, OS induced by liquid storage caused damage in sperm quality. When OS-caused damage is beyond the capability of spermatozoa to tolerate, the damage in mitochondrial functionality occurs, accompanied with the increasing number of dead spermatozoa due to the lack of energy. The results of the present study agree with the reported evidence that OS induced by excessive ROS is the main cause that provokes a continuous decline in sperm quality and functionality in liquid stored semen [29].

To overcome the detrimental effects of OS on semen storage, various antioxidants were added into the semen extender, and their protective effect on sperm quality was observed [11,33,34]. Furthermore, our previous investigation demonstrated that antioxidants in seminal plasma are directly involved in cryotolerance of boar spermatozoa by playing against OS induced by the freezing and thawing process [4]. By taking advantage of the previous findings, in the present study, we measured antioxidants and oxidants in boar semen to make a better understanding of changes in seminal quality during liquid storage at 17 °C. Under our experimental conditions, TAC and MDA levels significantly decreased in SF with storage time, being the lowest on day six and day seven. As is known, the TAC consists of low-molecular non-enzymatic antioxidants [35], originating from seminal plasma and commercial extender. MDA is the main product of lipid peroxidation. The evidence showing that the addition of 25% SP in boar semen increased the TAC level but decreased the MDA content and ROS production [36] indicates an interaction between TAC and lipid peroxidation. In the present study, the continuous decline in the SF TAC during storage suggests its protective role against oxidative stress, which could be confirmed by the decrease in the SF MDA content and intracellular ROS and TOS levels after four days of storage. In addition, antioxidants and oxidants in spermatozoa were determined. A declining trend of intracellular oxidant molecules in terms of TOS was observed, and the lowest value was observed on days five and six. The increase in sperm SOD activity during the first three days could be due to the increase in oxidative stress in terms of intracellular ROS and TOS levels, as the spermatozoa themselves have an enzymatic antioxidant system to respond to OS induced by storage. However, the antioxidants in spermatozoa are very limited due to the distribution and space of cytoplasm [37]. With increased storage time, the intracellular ROS accumulate to an amount beyond the scavenging capacity of intracellular antioxidants, breaking down the redox balance. In addition, the number of viable spermatozoa decreased with storage time, resulting in a reduction in SOD-like activity. Those results could explain the decrease in sperm SOD activity on day four. After four days of storage, the sperm AMPK was significantly activated. The increase in sperm SOD-like activity after four days of storage may be due to the activation of the sperm AMPK, which is suggested to promote the antioxidants in boar spermatozoa [33]. SOD scavenges O^2-•^ [12], and its supplementation into freezing medium was demonstrated to be beneficial to the cryosurvival of Black Bengal buck spermatozoa [38]. The results of the present study indicate the opposing roles between antioxidants and oxidants in spermatozoa and their surrounding environment during storage.

The AMPK plays a vital role in balancing cell metabolism and was first found in mammalian sperm in 2012 [14,15]. The activation of the AMPK was found to exert a positive effect on sperm quality and functionality, while the inhibition of the AMPK imposed a negative impact [14,15,17,18]. The phosphorylated AMPK is not detected in fresh boar semen, while liquid storage at 17 °C increases AMPK phosphorylation in a time-dependent manner, with the highest level being observed on day seven [39]. Similarly, in the present study, the p-AMPK increased significantly with storage time from day two to seven. However, a higher p-AMPK level was observed on day one compared to that at days two and three, which could be due to the higher level of intracellular ROS at day 1, as mentioned before. It has been demonstrated that the increase in the AMP/ATP ratio activates the AMPK, which upregulates the ATP-generating catabolic pathway and reduces the energy-consuming anabolic pathway [40]. In this study, a significant increase in the AMP/ATP ratio was observed after three days of storage, which corresponded to the elevated p-AMPK level. The notable increase in the AMP/ATP ratio from day one to day two could be attributed to the higher level of intracellular ROS production on day one, resulting in the activation of the AMPK at the beginning of the storage period. With increased storage time, increasing the p-AMPK levels may promote catabolic metabolism to produce more ATP; thus, the AMP/ATP ratio decreased, as observed after day four.

To evaluate the effect of antioxidants and oxidants in boar spermatozoa and SF on the sperm quality and activation of the AMPK, correlation analyses were performed. Sperm viability during liquid storage showed a positive correlation with SF TAC. The TAC measures low-molecular substances with antioxidant properties, such as ascorbic acid, uric acid, a-tocopherol, and glutathione [35], which are inserted into the cell membrane structure or bind to plasma membrane as an ROS scavenger [41]. As is reported, the TAC in seminal plasma relates to boar sperm survival [42]. The results of the present study confirmed the beneficial action of the TAC in maintaining boar sperm viability. Moreover, sperm viability was correlated to SF MDA, sperm TOS, and AMP/ATP ratios without statistical significance. The evidence that the addition of rosmarinic acid and resveratrol into boar semen extender favors boar sperm motility, acrosome, and plasma membrane integrity by improving TAC and SOD levels and reducing MDA contents [43,44], thus indicating the negative effect of MDA on sperm quality. Intracellular TOS levels may exert a negative effect on sperm viability, as is reported wherein boar spermatozoa with a lower plasma membrane integrity are accompanied by a higher TOS level [45]. ATP production by mitochondria is essential to maintaining sperm quality. The supplementation of proline into boar semen improves sperm quality, as well as ATP levels and redox homeostasis [46]. Once OS are generated, the synthesis of ATP decreases and ROS production increases [47], which in turn imposes a detrimental effect on sperm quality. Correlation analysis between antioxidants and oxidants was performed, as well as AMPK activation. The p-AMPK level was significantly correlated to SF MDA. A weak correlation was found between the AMP/ATP ratio and the SF TAC. The results suggest a possible association between the AMPK activation of boar spermatozoa and SF MDA, as well as the potential effect of the SF TAC on the AMP/ATP ratio. In somatic cells, cell stimulus that increases cAMP levels could promote sperm AMPK activation through cAMP degradation to AMP by phosphodiesterases [22]. Apart from sAC, cAMP, and the PKA pathway [14,16,21], OS, as a stress stimulus, could be another factor that activates the AMPK in boar spermatozoa. During storage, spermatozoa take in nutrients in a temperature-dependent manner from their surrounding environment to support basic biological process. Energy stress induces AMPK activation, which inhibits anabolic activity and simultaneously promotes catabolic activity to produce more ATP for cell survival [48]. ATP synthesis increases ROS production, which induces OS when levels exceed the antioxidant defenses. Therefore, the AMPK, as an energy sensor, may play an important role in maintaining redox balance.

To confirm the links between antioxidants and oxidants, and the activation of the sperm AMPK, H_2_O_2_ was employed to induce OS and the p-AMPK levels were determined. Meanwhile, antioxidant and oxidant indicators that were greatly influenced by liquid storage were selected to be measured. Treatment with H_2_O_2_ significantly damaged sperm quality, increased levels of oxidants, and decreased levels of antioxidants. Significantly higher p-AMPK and AMP/ATP ratio levels were observed when treated with different concentrations of H_2_O_2_. The results indicate that OS induced by H_2_O_2_ treatment promotes AMPK activation. It has been reported that lipid peroxidation caused by ROS acts as a stress stimulator in the plasma membrane by playing roles in AMPK activation [49]. Furthermore, ochratoxin A exposure to boar spermatozoa increases ROS production, which activates the AMPK [50]. However, the question surrounding how OS is involved in AMPK activation remains unanswered. In somatic cells, the AMPK can be activated by mitochondria-derived ROS via a possible secondary redox effect on ATP production [20]. The ATP level plays a core role in AMPK activation, which balances cellular energetic metabolism and exerts a positive impact on sperm quality [13]. In return, the activation of the AMPK by metformin increases the intracellular ATP level, resulting in a better boar sperm quality [51]. Thus, a similar signaling pathway leading to AMPK activation may occur in boar spermatozoa, as in somatic cells.

The final question regarding how the activated AMPK influences sperm quality remains. It has been reported that the addition of caffeic acid phenethyl ester and rosmarinic acid in boar semen improves sperm quality and promotes antioxidant capacity, possibly through affecting AMPK activity [33,52]. Further evidence in mouse embryonic fibroblasts and skeletal muscle suggests that the activation of the AMPK induces a peroxisome proliferator-activated receptor gamma coactivator-1α (PGC-1α)-dependent antioxidant response [53,54]. In addition, the AMPK may function through maintaining lactate and ATP levels [18]. More studies are required to provide direct evidence.

## 5. Conclusions

In summary, the present study demonstrates that boar spermatozoa liquid stored at 17 °C undergoes OS, which provokes damage in sperm quality. The antioxidants and oxidants in boar spermatozoa and their surrounding environment can be influenced by liquid storage. When oxidant levels predominate over antioxidant defenses, boar spermatozoa suffer from OS, which promotes the phosphorylation of the AMPK. The results suggest that antioxidants and oxidants are associated with AMPK activation.

## Figures and Tables

**Table 1:** Boar sperm quality and functionality during liquid storage at 17 °C. Data (mean ± SEM) from 25 ejaculates of 18 Duroc boars.

	Liquid Storage Time at 17 °C (d)						
	1	2	3	4	5	6	7
Total Motility (%)	71.92 ± 3.87	58.36 ± 4.85	51.96 ± 5.82	51.80 ± 5.85	57.92 ± 5.65	57.96 ± 5.48	54.60 ± 5.14
Progressive Motility (%)	45.44 ± 3.06	40.20 ± 3.60	35.96 ± 4.12	34.32 ± 4.05	37.60 ± 3.62	39.12 ± 3.92	35.60 ± 3.37
Viability (%)	74.20 ± 1.24 **^ac^**	74.78 ± 1.25 **^a^**	74.51 ± 1.22 **^a^**	70.28 ± 1.57 **^b^**	72.28 ± 1.55 **^ab^**	70.55 ± 1.24 **^bc^**	69.80 ± 1.52 **^b^**
Damaged acrosomal membrane in viable sperm (%)	1.54 ± 0.29	1.35 ± 0.32	1.12 ± 0.18	1.84 ± 0.38	1.76 ± 0.31	1.31 ± 0.22	1.38 ± 0.24
Intracellular ROS production (10^6^ Fluorescence unit/10^6^ viable sperm)	20.22 ± 1.72 **^a^**	7.33 ± 0.74 **^bc^**	11.92 ± 1.38 **^cd^**	10.88 ± 1.26 **^bcd^**	11.92 ± 0.94 **^ad^**	7.32 ± 0.53 **^b^**	6.81 ± 0.56 **^b^**
Mitochondrial membrane potential (%)	55.90 ± 6.84 **^ab^**	63.26 ± 5.05 **^a^**	48.36 ± 5.21 **^ab^**	38.93 ± 5.09 **^ab^**	60.00 ± 5.49 **^a^**	35.60 ± 5.59 **^b^**	40.87 ± 4.48 **^ab^**

Note: Letters ^a^, ^b^, ^c^ and ^d^ indicate differences among groups, *p* < 0.05.

## Data Availability

The data presented in this study are available in the article.

## Supplementary Materials

The following supporting information can be downloaded at: https://www.mdpi.com/article/10.3390/vetsci10030214/s1; Figure S1: WB full membrane for Figure 3; Figure S2: WB full membrane for Figure 6.
