# Supplementary figures and images for: Antioxidants and Oxidants in Boar Spermatozoa and Their Surrounding Environment Are Associated with AMPK Activation during Liquid Storage
Source: Vet Sci. 2023 Mar 10;10(3):214. doi: 10.3390/vetsci10030214 (PMC10056163; doi:10.3390/vetsci10030214)

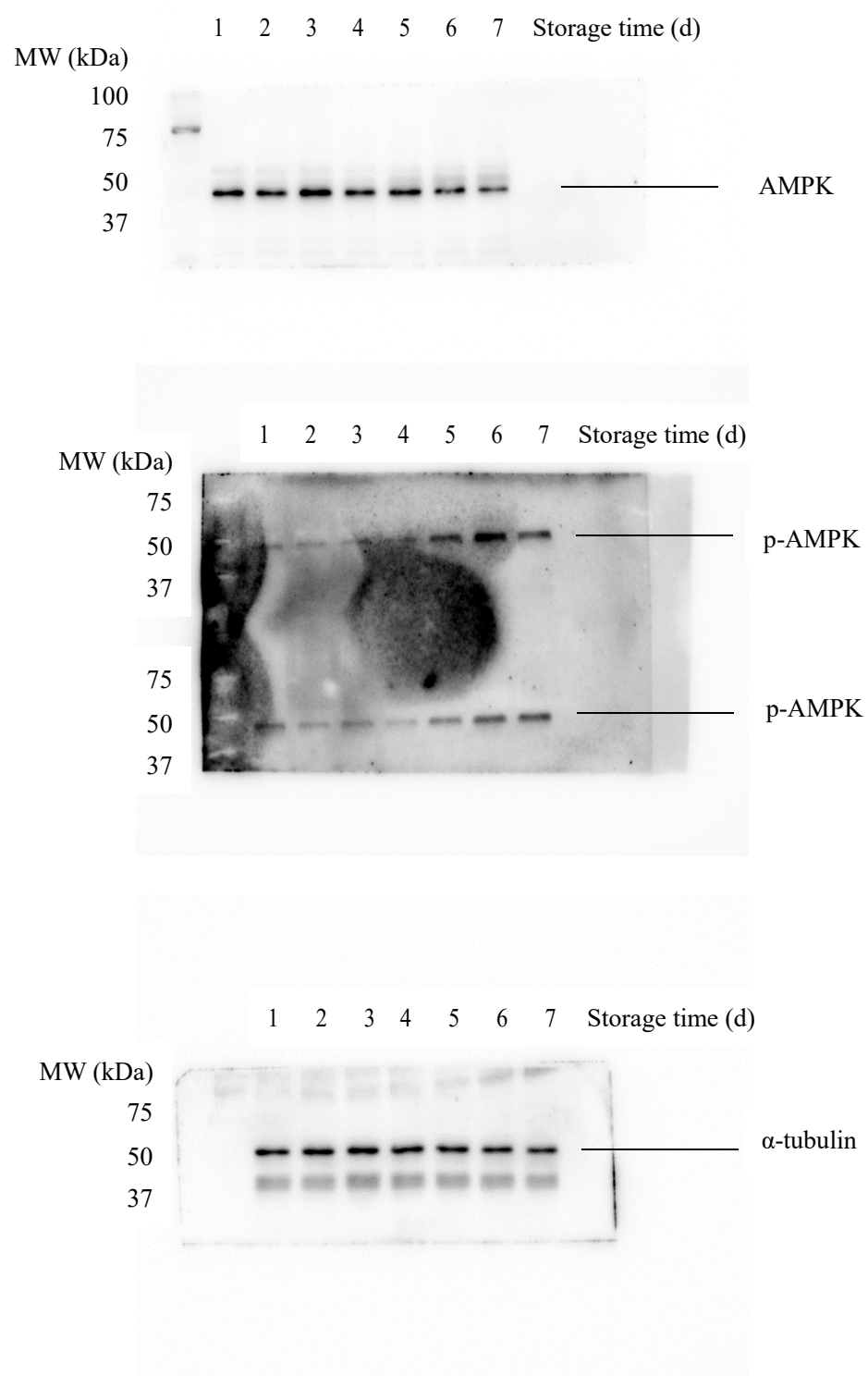

**Figure S1.** WB full membrane for Figures 3.

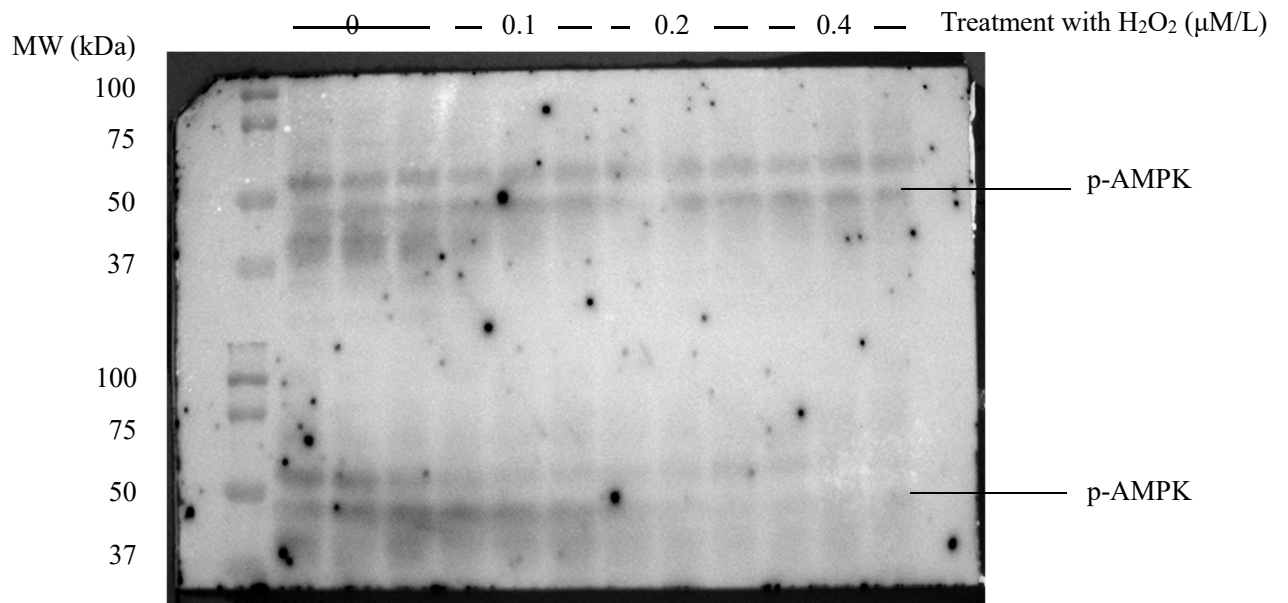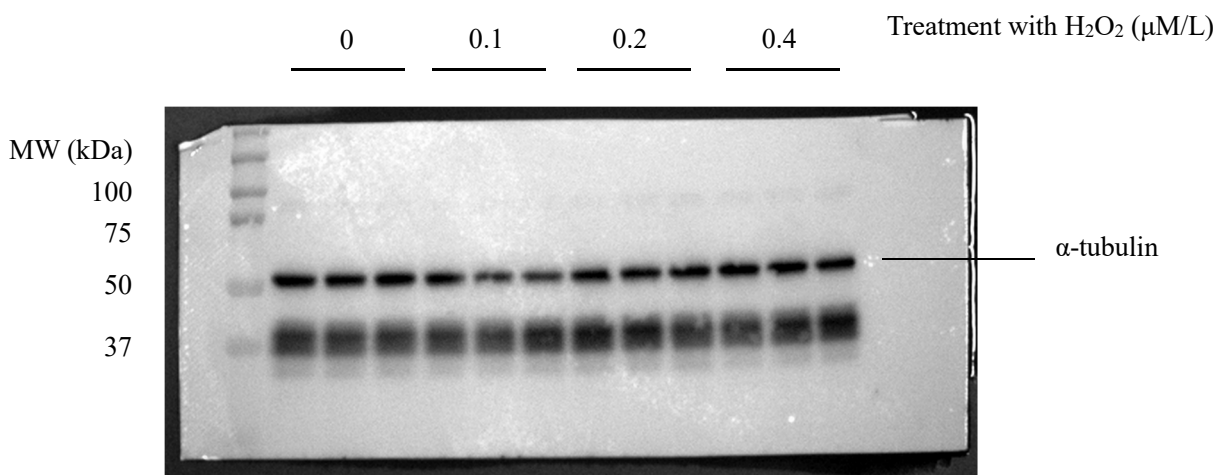

**Figure S2.** WB full membrane for Figures 6.

Supplement: Supplementary file 1 [file vetsci-10-00214-s001.zip › vetsci-2239534-supplementary.pdf]
